# Making a Correct Diagnosis of Glaucoma: Data From the EMGT

**DOI:** 10.1097/IJG.0000000000001342

**Published:** 2019-10-03

**Authors:** HannaMaria Öhnell, Boel Bengtsson, Anders Heijl

**Affiliations:** * Department of Clinical Sciences in Malmö, Ophthalmology, Lund University; † Department of Ophthalmology, Skåne University Hospital, Malmö, Sweden

**Keywords:** glaucoma, correct diagnosis, Glaucoma Hemifield Test, EMGT, perimetry

## Abstract

A correct diagnosis of glaucoma established at initial visits.

**Purpose:**

It has been suggested that a diagnosis of glaucoma cannot be certain until progression has been demonstrated. Our aim was to evaluate the correctness of a glaucoma diagnosis established after 2 initial visits.

**Patients and Methods:**

Patients included in the Early Manifest Glaucoma Trial (EMGT) who had continued follow-up for at least 15 years were included in this analysis. The patients had been recruited primarily through a population screening and were diagnosed with glaucoma if the Glaucoma Hemifield Test was outside normal limits in the same sector at two consecutive visits. A Glaucoma Hemifield Test classification of borderline was also diagnostic if corresponding optic disc findings were present. At least one of the following criteria had to be fulfilled during follow-up to confirm the initial diagnosis: (1) visual field progression in at least one eye according to the EMGT criterion; (2) development of manifest glaucoma in an initially ineligible fellow eye; (3) optic disc progression in at least one eye; (4) optic disc hemorrhages in at least 1 eye.

**Results:**

Of the 255 patients included in the EMGT, 117 were followed for at least 15 years, representing 147 eyes eligible for our study. During follow-up, 134 eyes (91%) showed visual field progression, and, of the remaining 13 eyes, only 4 (3%) did not fulfill any of the criteria to confirm the diagnosis.

**Conclusions:**

A diagnosis made applying strict criteria to 2 initial visual field tests, supported by optic disc findings if visual field findings were borderline, was almost always correct.

During the mid-19th century, our current understanding of glaucoma began to emerge, assisted by the construction of the ophthalmoscope and the tonometer.^1^–^4^ A diagnosis of primary open-angle glaucoma was made when an eye was identified with elevated intraocular pressure (IOP).^1^,^5^–^8^ This seemed particularly attractive one century later when Wolfgang Leydhecker was the first to define precise limits for normal IOP based on tonometry of large numbers of individuals from the general population.^9^ However, it was not long before the results of the first population study on glaucoma were published by Hollows and Graham,^10^ which showed that a substantial proportion of patients with glaucoma had pressures within the statistically normal limits. Those investigators also noted that a large percentage of individuals with IOP above the normal limits had no diagnostic signs of glaucoma damage and that finding was soon confirmed in another population study performed by Strömberg and Linnér.^11^ Numerous subsequent population studies have corroborated the mentioned observations,^12^ and it has now been accepted for decades that a diagnosis of open-angle glaucoma must be based on identification of signs of glaucoma damage rather than on tonometry alone.

Still, the risk of having glaucoma increases exponentially with higher IOP values.^13^–^15^ At least in the western world, most patients with glaucoma are also diagnosed with elevated IOP, and the suspicion of glaucoma has been based on increased IOP revealed by routine tonometry. Other possibilities for addressing the same question include identifying a potentially abnormal optic disc or a positive family history of glaucoma.

Once there is a suspicion of glaucoma, the clinical examination will focus on finding signs of glaucoma damage, structural or functional, and perhaps also risk factors for glaucoma, such as exfoliation syndrome or optic disc hemorrhages. False positives are rather common when judging optic discs,^16^ especially with larger discs,^17^ and also during visual field testing. A significant number of patients do not perform well at their first perimetry test, and the existence of perimetric learning is well established.^18^–^20^ If the glaucoma is in moderate or advanced stages, structural and functional changes are usually very clear and in agreement, and accurate diagnosis can be made immediately and without the need to consider aspects such as confirmation of, for example, perimetric findings with a second test. In earlier stages of the disease, the findings are less convincing. Also, when diagnosing glaucoma damage in patients who are being followed due to ocular hypertension, it is necessary to confirm findings at several consecutive visits to avoid a very large percentage of false-positive diagnoses.^21^–^23^

It is often stated that in order to be absolutely sure of a glaucoma diagnosis, it is necessary to document progression.^24^,^25^ This is indeed a suitable way of avoiding false-positive diagnoses, which result in unnecessary treatment and reduction of quality of life.^26^,^27^ The glaucomatous disease process is often slow, and it typically takes years to find clear signs of structural or functional progression.^28^ Glaucoma treatment slows disease progression,^29^–^31^ and hence it is probable that neither patients nor physicians will be willing to withhold treatment if the diagnosis is or is very likely to be correct. Thus, the clinical standard is to start treatment as soon as the diagnosis is made, and not to wait for signs of progression.

Considering the above, it is therefore of interest to determine how often a diagnosis of open-angle glaucoma made without follow-up is correct or, perhaps of even greater interest, how often it is incorrect. The answer in that context depends on the methods used to make the diagnosis. We have access to data from the Early Manifest Glaucoma Trial (EMGT), which gave us the opportunity to study this issue.^32^ The majority of EMGT patients were identified through population screening aimed at detecting previously undiagnosed early to moderate glaucoma. Patients entered the trial between 1994 and 1997, and prospective protocol-based follow-up continued until the end of 2013. This long prospective follow-up provided an unusual possibility to determine whether the initial glaucoma diagnoses made without follow-up and proof of progression were reliable or incorrect. Patients were identified by a suspicious optic disc or retinal nerve fiber layer findings in fundus photographs, or elevated IOP. The diagnosis was based on repeated demonstration of visual field defects meeting predetermined criteria, and also on optic disc appearance if visual field findings were borderline.

Our aim was to investigate whether patients could be correctly diagnosed with glaucoma during two initial visits, or if signs of progression or disease activity are necessary for a reasonably specific diagnosis.

## PATIENTS AND METHODS

The EMGT (National institutes of Health ClinicalTrials.gov identifier NCT00000132; date of registration September 23, 1999) was a prospective, randomized, controlled treatment trial aimed at studying the effect of intraocular pressure reduction on glaucoma progression. This objective was achieved by randomizing patients who had newly detected glaucoma with field loss either to a fixed treatment protocol (topical betaxolol plus argon laser trabeculoplasty) or to no treatment. In the respective groups, the treatment or lack of treatment was maintained unchanged as long as protocol-defined progression had not occurred. The study was conducted according to the tenets of the Declaration of Helsinki and was approved by the Ethics Committee of the University of Lund, Sweden, and by the Committee on Research Involving Human Subjects of the State University at Stony Brook, New York. All patients provided informed consent.

The majority of patients were recruited through a large population-based screening of 44,000 subjects aged 55 to 79 years in 2 cities in southern Sweden. Screening examinations included Goldmann applanation tonometry and fundus photography, but not visual field testing. Subjects who screened positive by an IOP>25 mm Hg, suspicion of glaucomatous optic disc changes, optic disc hemorrhages, retinal nerve fiber layer defects or a family history of glaucoma among siblings were called back for a full clinical postscreening examination including Humphrey 24-2 full-threshold visual field tests.

A diagnosis of glaucoma was made or rejected at 2 postscreening visits. The criterion was repeatable visual field defects that had to be compatible with glaucoma and not explained by other causes. Two reliable fields were required with a Glaucoma Hemifield Test (GHT) classification of “outside normal limits,” with the significantly depressed test point locations triggering the criterion present in the same sector of 10 possible GHT sectors in both tests.^33^ An eye was also considered glaucomatous and eligible if the GHT classification was “borderline” at one or both of the postscreening visits, still requiring the depressed test points to be located in the same GHT sector in both tests. However, if the GHT classification was borderline, glaucomatous optic disc changes were also required in an area corresponding to that of the field loss. The level of the IOP was not considered for diagnosis. Visual fields with a GHT classified as “abnormally high sensitivity” were regarded as unreliable.

All of the patients with newly detected glaucoma were not eligible for inclusion in the EMGT. Advanced visual field loss was an exclusion criterion, and therefore patients with a glaucomatous eye with a mean deviation value worse than −16 dB were ineligible. Also, the maximum permitted mean untreated prestudy IOP was 30 mm Hg, and no single IOP reading of >35 mm Hg was allowed for eligibility.

Thus, the EMGT patient cohort analyzed in the present study consisted of a group of patients who had earlier disease compared with clinically diagnosed patients, for these 3 reasons: (1) patients with advanced damage were not eligible; (2) patients with mean IOP >30 mm Hg were not eligible; (3) almost all patients came from a large population-based screening.

The trial participants had regular follow-up every 3 months until 2002, at which time the primary question addressed by the EMGT had been answered. Computerized threshold perimetry (Humphrey 30-2 full-threshold tests) was performed at each visit, and optic disc photography was conducted every 6 months. Thereafter, a minority of the patients had visits every 6 months, whereas the great majority continued follow-up at 3-month intervals until 2005, when the frequency of follow-up visits was tailored to the need of the individual patient, although with a minimum of one protocol visit per year. The maximum follow-up period was 25 years. In 2005, the visual field testing protocol was changed from the older full-threshold 30-2 test to the Swedish Interactive Threshold Algorithm (SITA) Fast 30-2.

Glaucoma progression was defined by predetermined EMGT visual field criteria. Definite field progression was defined as at least three identical points in the 30-2 or 24-2 test point patterns showing significant deterioration in glaucoma change probability maps in 3 consecutive tests. Tentative progression was defined as at least 3 identical points showing significant deterioration in glaucoma change probability maps in 2 consecutive tests. When this was found, the subject was called back for an extra examination including visual field testing 1 month later.^32^ The definite field progression criterion has been shown to be both sensitive and specific for progression.^34^,^35^ In the current analysis, only visual field progression that was caused by glaucoma and was sustainable throughout the rest of the series was regarded as glaucomatous field progression.

In the present project, we performed new disc analyses to include all available data, and a detailed description of these analyses has been published.^28^ Briefly, in the first step 3 graders independently compared the 3-month photograph of each eye (rather than the baseline photograph to avoid short-term changes induced by IOP reduction)^36^,^37^ with the last photograph obtained using the same camera technique as in 2005. Temporal order was masked. One grader (H.M.Ö.) evaluated the full series of photographs of each eye to detect any progression that might have been missed in the pair analyses. Optic disc changes regarded as signs of progression were changes in the course of vessels in the optic disc or a change in the neuroretinal rim such as notching. During this session, all photographed optic disc hemorrhages were also noted. Discrepancies were resolved by consensus. The assessment of the photographs during the final years had to be done unmasked with regard to temporal order and was performed by 2 graders only (H.M.Ö., A.H.).

Our aim in the current study was to ascertain whether the initial diagnosis of glaucoma would prove to be correct in the long run. To achieve this, we investigated each patient for functional or structural disease progression or disc hemorrhages in both eyes. In as much as glaucoma progression can be very slow, only patients with at least 15 years of follow-up were included in the current analyses.

We applied the following 4 criteria to confirm that a patient had been correctly diagnosed with glaucoma; fulfilling one of them was considered enough for confirmation.

1. Definite visual field progression in at least one eye according to the predetermined EMGT criteria.
2. Development of manifest glaucoma in an initially ineligible fellow eye, applying the same definition of glaucoma as was used for inclusion in the EMGT.
3. Optic disc progression in at least one eye, as described above.
4. Occurrence of at least one optic disc hemorrhage in either eye, as described above.

## RESULTS

**FIGURE 1:**
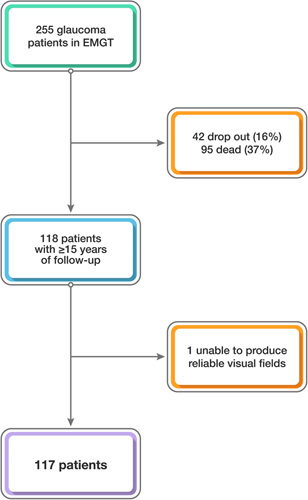
Flow-chart for glaucoma patients from the Early Manifest Glaucoma Trial (EMGT) included in the current analysis.

Of the initial 255 randomized subjects, 117 had been followed in the EMGT for at least 15 years (median: 19.7 y) and represented 147 eyes eligible for the present study (Fig. 1). Baseline characteristics are listed in Table 1. The 117 patients were identified for postscreening examinations and subsequent inclusion in the EMGT due to a suspicious optic disc in 84% of cases, optic disc hemorrhage in 19%, IOP above 25 mm Hg in 13% and other reasons in 20%.

**TABLE 1:**
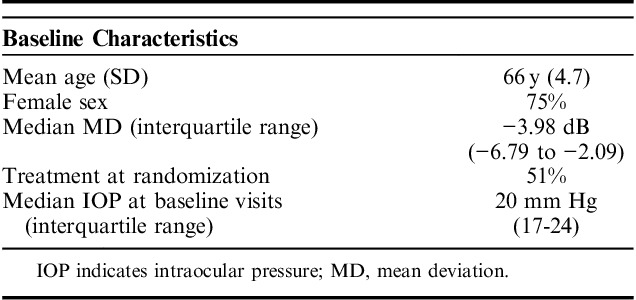
Baseline Characteristics of 147 Study Eyes in 117 Patients With Follow-up of ≥15 Years in the Early Manifest Glaucoma Trial

Thirteen eyes (9%) of 13 patients had not shown definite and sustainable glaucomatous visual field progression at the end of the follow-up. Among these 13 eyes, 3 eyes had been classified as progressing in earlier EMGT intent to treat analyses: the visual field criterion had been triggered by a stroke in one of these cases and by a droopy eyelid in a second case, and in the third eye the field progression was not sustained. Table 2 outlines fulfillment of the 4 criteria necessary for the 13 patients without visual field progression to be considered correctly diagnosed. In 5 of these 13 patients (nos. 506, 614, 622, 678, 839), the fellow eye showed glaucoma progression or incident manifest glaucoma with field loss. Two eyes (1 each in patients 679 and 839) showed optic disc progression. Optic disc hemorrhages were seen in the study eyes without field progression in 4 patients (506, 537, 622, and 673) and in the fellow eye in 1 patient (670).

**TABLE 2:**
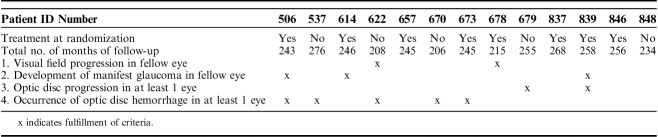
Fulfillment of the 4 Criteria to Confirm the Glaucoma Diagnosis in the 13 Eyes of 13 Patients in the Early Manifest Glaucoma Trial Showing No Visual Field Progression Despite ≥15 Years of Follow-up

**FIGURE 2:**
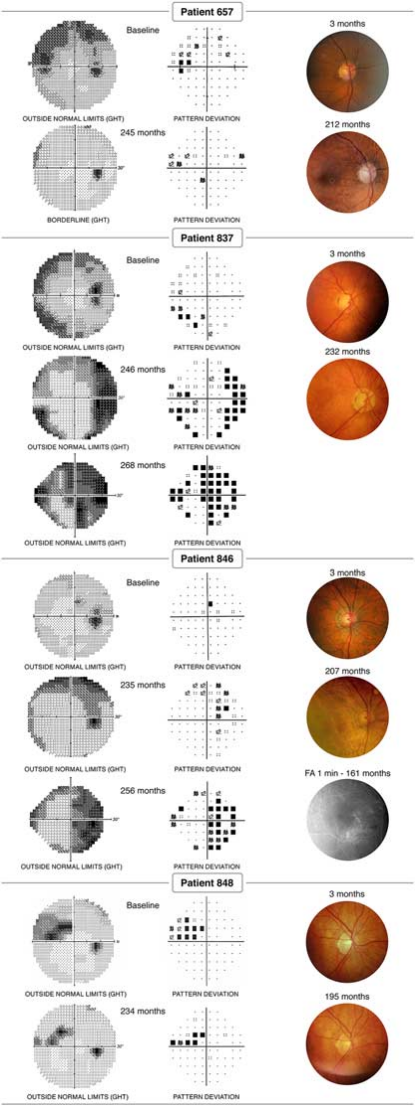
Visual fields and optic disc photographs of eyes in 4 patients for whom the glaucoma diagnosis could not be confirmed after ≥15 years of follow-up. For patient 657, there was no other reasonable explanation for the field defects. Patient 837 had failing general health during the final visual field examination, and hence the preceding field is also included; this patient also had no other apparent reason for the field defect. For patient 846, a fluorescein angiogram (FA 1 min) with a perceivable pigment epitheliopathy is included here; this patient’s final field was also worse than previous fields, albeit for uncertain reasons, and thus the last 2 fields are shown. Patient 848 had a small retinochoroiditis scar that can explain the field defects.

Thus, 4 eyes (in patients 837, 848, 846, and 657) remained that fulfilled none of the criteria. Visual fields and optic disc photographs of these four eyes recorded at the first baseline visit and the final visit are shown in Figure 2 and can be summarized as follows:

- One eye (in patient 837) had small but sustainable field defects that increased somewhat and showed tentative EMGT visual field progression at the last follow-up visit, 18 years after baseline. We believe, but cannot be sure, that this patient was correctly diagnosed at baseline.
- One eye (in patient 848) represented a faulty diagnosis. This eye had a very clear and substantial paracentral field defect that remained unchanged over time, probably due to a small retinochoroiditis that was missed at baseline. The disc showed saucerization only and did not change over time.
- One eye (in patient 846) exhibited a sustainable field defect, although usually at only a single test point location and occasionally surrounded with shallower and less significant possible defects. The fellow eye developed macular changes many years later. Images of the study eye are included in angiographies performed at that time and reveal that the study eye had a retinal pigment epitheliopathy in an area corresponding to the field defect. We believe that this eye also represents a faulty diagnosis.
- One eye (in patient 657) initially had apparent early field loss that decreased over time, but small abnormalities also remained in the probability maps over time, although these were not observed at every test but always in the same area. This eye only had suspicious notching of the optic disc with no visible progression. It is nearly a philosophical question whether this should or should not be regarded as glaucoma.

## DISCUSSION

In our analysis of 147 EMGT study eyes with follow-up times of at least 15 years, we found 13 eyes (9%) with no formal definite visual field progression according to the predetermined progression criteria. Considering these 13 eyes, all but four of the patients (3%) displayed other signs of glaucoma: progression of glaucomatous field defects or incident glaucoma in the fellow eye, occurrence of optic disc hemorrhages, or documented optic disc progression. In only 2 of the 13 eyes (1%) were we able to find any likely explanation for the perimetric findings other than glaucoma.

An advantage of this study is the long follow-up time with very low drop-out rates, which made it possible to document signs of progression or disease activity that in some cases became apparent only after many years. Another strength is the prospective design with protocol-controlled follow-up including regular acquisition of both visual field and structural data. No data were collected in the EMGT by use of modern imaging techniques because such instruments were not available at the time the trial was initiated. Furthermore, our data cannot be used to assess the sensitivity of the diagnostic criteria. Considering GHT, Sekhar et al^38^ have previously described sensitivity of 95%, and Susanna et al^39^ a sensitivity of 100%, but obviously sensitivities depend on the stage of glaucoma that is studied.

The need for a very long follow-up in order to be able to confirm the initial diagnosis due to the slow progression rate in some patients has been demonstrated also in other studies, for instance, the Collaborative Normal Tension Glaucoma Study, where 40% of participants had not shown signs of progression after 7 years of follow-up.^40^

Even though we had access to data that had been assembled systematically during a very long follow-up period, we cannot be entirely sure of the diagnosis for the 2 patients for whom we found no proof of glaucoma activity and no other findings that could explain the initial visual field loss that was required for eligibility. We do not know whether these patients represented false-positive diagnoses, or if they had eyes with nonprogressive glaucoma. Nonetheless, it can be concluded that, at most, only 4 patients (3%) were incorrectly diagnosed at baseline.

Diagnosing patients for inclusion in the EMGT differed somewhat from making diagnoses in ordinary clinical care. Most of the patients were identified through a population-based screening, and thus they had earlier stages of glaucoma compared with self-selected patients.^41^ Late-stage glaucoma cases with high IOP or severe visual field defects were also excluded. This advanced group of glaucoma cases seldom present any diagnostic difficulties. Therefore, it could be speculated that, in a clinical setting, the percentage of false-positive diagnoses might have been even smaller.

The screening procedure was based largely on identifying suspicious optic discs. Thus, it is possible that the screening for selection of patients did not identify subjects who had visual field defects with no detectable structural damage. In clinical care, a suspicious optic disc is obviously also a standard reason to consider the possibility of glaucoma and perform a full examination to confirm or reject such a diagnosis, and it is just as likely that glaucoma patients with small discs and early damage will be missed.

Study participants were almost entirely Caucasians. It is not possible to draw the conclusion from this data that the diagnostic criteria would perform similarly in other ethnic groups, but we do not believe that such differences are likely, as age-corrected normal perimetric threshold values do not differ among races.

Although only about half of the original EMGT cohort reached 15 years of follow-up, the majority (95/137) failed the checkpoint due to death. Among the 16% that dropped out most did so due to old age, moving to residential homes or suffering from other conditions that prevented study visits. It is highly unlikely that these patients would be incorrectly diagnosed to a larger extent than the patients that did fulfill 15 years of follow-up.

The present study considered patients who had no prior experience of perimetry and were newly diagnosed with glaucoma based on repeatable visual field defects with GHT results classified as outside normal limits in the same sector or as borderline with corresponding optic disc findings. A correct diagnosis could be made for at least 97% of those patients without any follow-up results. These findings indicate that a diagnosis is almost always correct when it is based on results from visual field testing using strict criteria for interpretation and backed up with structural findings if field findings are borderline. In short, with this approach, there is little risk of making a false-positive diagnosis.
